# Clinical, Radiological, and Genetic Profile of Spinocerebellar Ataxia 12: A Hospital-Based Cohort Analysis

**DOI:** 10.5334/tohm.686

**Published:** 2022-04-21

**Authors:** Valakunja Harikrishna Ganaraja, Vikram V. Holla, Albert Stezin, Nitish Kamble, Ravi Yadav, Meera Purushottam, Sanjeev Jain, Pramod Kumar Pal

**Affiliations:** 1 Department of Neurology, National Institute of Mental Health & Neurosciences (NIMHANS), Hosur Road, Bangalore-560029, Karnataka, IN; 2 Department of Clinical Neurosciences, National Institute of Mental Health & Neurosciences (NIMHANS), Hosur Road, Bangalore-560029, Karnataka, IN; 3 Molecular Genetics Laboratory, Department of Psychiatry, National Institute of Mental Health & Neurosciences (NIMHANS), Hosur Road, Bangalore-560029, Karnataka, IN

**Keywords:** Spinocerebellar ataxia type 12, Tremors, Ataxia, Trinucleotide repeats

## Abstract

**Introduction:**

Spinocerebellar ataxia type-12 (SCA12) is a rare form of SCA, most commonly reported from the Indian *Agarwal* and related families. In this study we describe the clinical, genetic, and radiological characteristics of a sizeable cohort of genetically proven SCA12.

**Methods:**

A retrospective chart-review of the genetically confirmed SCA12 patients from our centre. The demographic, clinical, and investigation findings were reviewed. Correlation of expanded repeats length with various demographic and clinical features were studied.

**Results:**

A total of 49 patients (34 males, 42 families) were included of which 79.6% belonged to *Agarwal* community. The mean age at onset and age at presentation were 46.38 ± 11.7 years and 53.16 ± 12.78 years respectively. The most common initial symptom was tremor (73.5%), followed by ataxia (18.4%). At presentation, 95.9% of the patients had tremor with predominant distribution in the bilateral upper limbs (85.7%). At presentation, 73.5% of patients had ataxia and 22.4% had cognitive dysfunction. The mean CAG repeat length in *PPP2R2B* in the expanded allele was 53.26 ± 6.10 (40–72). The lowest pathogenic expanded repeat sizes in *PPP2R2B* recorded in our cohort was 40 & 42 repeats from two patients with a consistent clinical phenotype. Another unusual phenotype was the presence of prominent myoclonus. There was no significant correlation between the age at onset of symptoms and the repeat size of CAG repeat.

**Conclusion:**

SCA12 is not confined to a single ethnicity. Upper limb tremor and ataxia were the most common presentation. Unusual presentation may cause diagnostic confusion especially when recorded in patients from non-*Aggarwal* families.

## Introduction

Spinocerebellar ataxia type 12 (SCA12) is an uncommon forms of autosomal dominant spinocerebellar ataxia which is most commonly reported from India [1]. It is caused by an unstable expansion of CAG trinucleotide repeat in the 5’ untranslated region of gene protein phosphatase 2 regulatory subunit Bb (*PPP2R2B*) on the long arm of chromosome 5 (5q32). This gene encodes a regulatory subunit of ubiquitous serine/threonine phosphatase enzyme PP2A that regulate cell cycle, cell development, and various signaling pathways [2].

Being a rare ataxic disorder, there is a huge gap in the present understanding of clinical phenotypes and community specificity of this disorder. At present it is understood that this form of degenerative ataxia is confined to the *Agarwal* community in India [3,4,5]. There is only limited data on the prevalence of SCA12 patients from non-*Agarwal* community. The cut-off for pathogenic trinucleotide repeats length in SCA12 is also unclear. In this article, we describe our experience in working with a large cohort of genetically confirmed SCA12. We also report unusual CAG repeat size and phenotypes in our SCA12 cohort.

## Methods

This study was performed at the National Institute of Mental Health and Neurosciences (NIMHANS), Bengaluru as a retrospective chart review. This study was approved by the Institute Ethics Committee [No.NIMH/DO/DEAN(Basic Science)/2020-21] and informed consent for use of data was obtained from the patients.

The diagnosis of SCA12 was established on the basis of clinical features and on the presence repeat length of ≥ 40 CAG in the 5’ untranslated region of *PPP2R2B* gene. The demographic, clinical, imaging and genetic details of consenting patients were retrieved from the hospital records and imaging databases. All patients were examined by a senior movement disorder specialist (PKP).

The retrieved clinical data included details such as age, age at onset, clinical presentation and duration of symptoms, presence of family history, ethnicity, and presence of neurological signs. In case of unusual phenotypes, the patient’s records were reviewed along with archived clinical examination videos. Brain imagings were retrieved from hospital PACS system and visual observations were recorded. All the findings were entered in a pre-structured proforma and was used for analysis.

### Statistical analysis

The data was expressed as mean, median, standard deviation, and range for continuous variables and in frequency and percentage for categorical variables. After the normality of data was established, either Pearson or Spearman’s correlation was applied to study the association/correlation of expanded CAG repeat length with demographic and clinical features. A threshold value of p < 0.05 after correction for multiple comparison was considered as significant. All statistical analysis was done by SPSS version 23.

## Results

### Demographic data

Forty-nine patients (34 males) from 42 different families with genetically proven SCA12 were included in this analysis. The mean age at onset of symptoms was 46.38 ± 11.7 years; the mean age at presentation to hospital was 53.16 ± 12.78 years, and the mean duration of illness was 9.20 ± 9.5 years. Family history of SCA12 was present in 93.8% of patients.

Thirty-nine patients (79.6%) belonged to the *Agarwal* community, whereas 5 patients (10.2%) were from non-*Agarwal* community. Nearly 70% of patients were residing in the north-eastern part of India (Orissa, West Bengal, Bihar, Jharkhand, Delhi, Rajasthan, Uttar Pradesh, Uttarakhand and Assam) while 18% of the patients belonged to southern part of India. Details are given in ***Table 1***.

**Table 1:** Demographic profile of patients with SCA 12.


	TOTAL NUMBER	PERCENTAGE

Total number of patients	49	100%

Male: Female	34:15	

Place of residence		

Orissa	10	20%

West Bengal	10	20%

Karnataka	7	14%

Bihar	4	8%

Jharkhand	4	8%

Madhya Pradesh	3	6%

Maharashtra	2	4%

Assam	2	4%

Tamil Nadu	2	4%

Chhattisgarh	1	2%

Delhi	1	2%

Rajasthan	1	2%

Uttar Pradesh	1	2%

Uttarakhand	1	2%

Community		

*Agarwal*	39	79.6%

Unclear/not disclosed	5	10.2%

Non-*Agarwal*	5	10.2%

Urban: Rural	42:7	

Positive family history	46	93.8%

Co-morbidities		

DM	13	26.5%

HTN	13	26.5%

Hypothyroidism	5	10.2%

IHD	2	4%

Asthma	1	2%


Abbreviations: DM- diabetes mellitus, HTN- hypertension, IHD- ischemic heart disease.

### Clinical features

**Figure 1:**
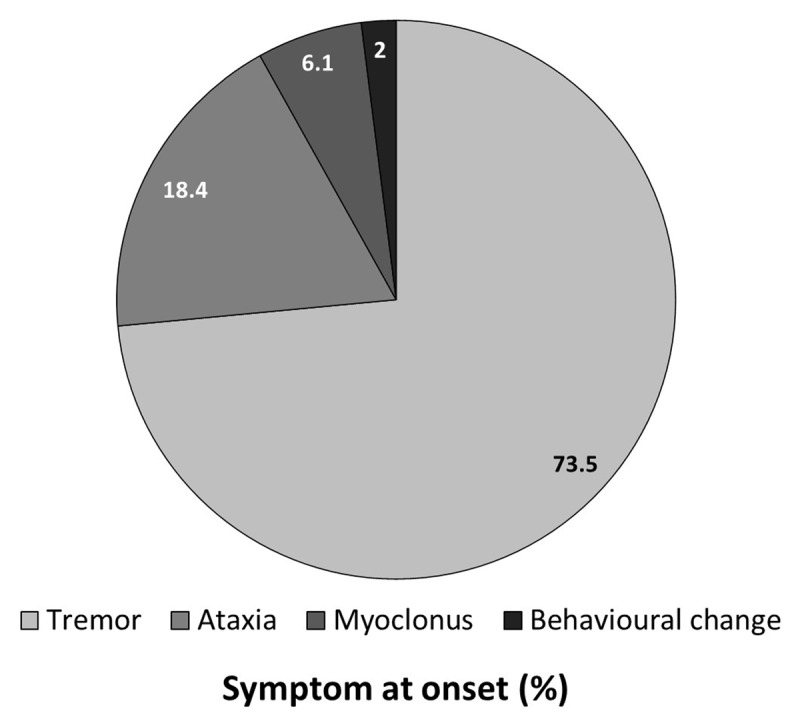
Symptom at onset of disease among the patients with SCA12.

**Figure 2:**
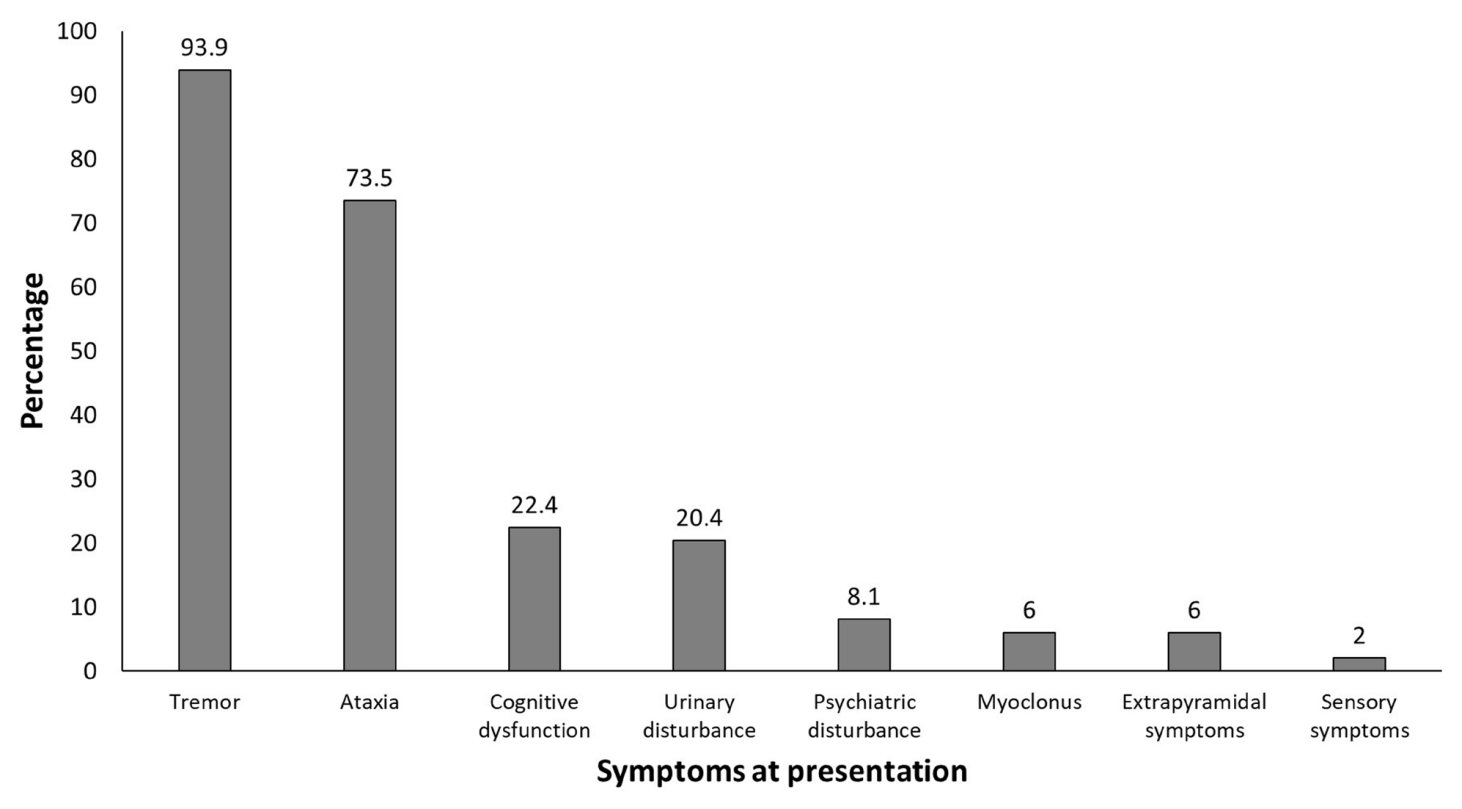
Symptoms at presentation among the patients with SCA12.

The most common initial symptom was tremor (36 patients, 73.5%) followed by ataxia (9 patients, 18.4%) and myoclonus (3 patients, 6.1%) (***Figure 1***). At the time of presentation to hospital, tremor was reported in 47 patients (95.9%) and ataxia in 36 patients (73.5%) (***Figure 2***). All subjects had right hand dominance.

### Tremor

Tremor was present in 47 patients (95.9%) at the time of presentation. The onset of tremor was in right upper limb in 26 patients (53.1%), left upper limb in 2 patients (4.1%), bilateral in 17 patients (34.7%) and affected the head in 2 (4.1%).

At the time of presentation (mean duration = 7.13 ± 4.28 years), tremor involved bilateral upper limbs in 42 patients (85.7%) and remained isolated in five (10.2%) patients (right upper limb: 2 patients, left upper limb: 1 patient, head tremor:2 patients). Among those with bilateral tremor at presentation, it was bilaterally symmetric in 35 patients (71.4%) and right more than left in the remaining 7 patients (14.3%).

Head tremor was noted in 27 patients (55.1%), voice tremor in 21 patients (42.8%), jaw tremor in 5 patients (10.2%) and tongue tremor in 5 patients (10.2%). Based on the activation condition, postural tremor was present in 43 patients (87.7%), intentional type in 28 patients (57.1%) and at rest in 18 patients (36.7%). Combination of rest and postural tremors were observed in 16 patients (32.6%). We did not observe leg tremors in this cohort.

### Ataxia

Thirty-six patients (73.5%) had symptoms of imbalance at the time of presentation with a mean duration of symptoms for 4.88 ± 3.86 years. However, on clinical examination, 44 patients (89.8%) had cerebellar signs. Ataxia and impaired tandem gait were present in 44 patients (89.9%), dysmetria in 37 patients (75.5%), dyssenergia and dysdiadokinesia in 33 patients (67.3%) each. Among these patients with SCA-12, 41 patients (83.7%) were ambulant at the time of presentation with the mean duration of illness of 8.7 ± 5.1 years whereas, the remaining eight patients (16.3%) needed walking aid for ambulation with mean duration of illness of 5.8 ± 3.38 years. The data on severity of ataxia (measured by clinical scales) was not uniformly available in all patients of the cohort hence not studied further.

### Other neurological signs

Ocular motor abnormalities were present as slow saccades in 13 patients (26.5%) and broken pursuits in 14 patients (28.6%). Pyramidal signs were present in the form of hyperreflexia (23 patients, 46.9%) and spasticity (9 patients, 18.4%). The extrapyramidal signs in the cohort included bradykinesia (15 patients, 30.6%), rigidity (7 patients, 14.2%) focal dystonia (2 patients, 4.1%) and generalised chorea (1 patient, 2%). These details are tabulated in ***Table 2***.

**Table 2:** Ataxic and non ataxic features of patients with SCA 12.


Age at onset:	46.38 ± 11.7 years	

Duration of illness:	9.20 ± 9.5 years	

Age at presentation:	53.16 ± 12.78 years	

Symptom at onset:	Total numbers	%

Tremors	36	73.5%

Ataxia	9	18.4%

Myoclonus	3	6.1%

Behavioural changes	1	2%

Symptom at presentation		

Tremors	47	95.9%

Ataxia	36	73.5%

Cognitive dysfunction	11	22.4%

Urinary disturbance	10	20.4%

Psychiatric disturbance	4	8.1%

Myoclonus	3	6%

Extrapyramidal symptoms	3	6%

Sensory Symptoms	1	2%

**Tremor**	47	95.9%

Duration	7.13 ± 4.28 years	

Side and site at onset		

Right UL	26	53.1%

Bilateral	17	34.7%

Left UL	2	4.1%

Head	2	4.1%

Side of appendicular tremor at presentation		

Bilateral –Symmetrical –Right>Left	42 –35 –7	85.7% –71.4% –14.3%

Right UL	2	4.1%

Head	2	4.1%

Left UL	1	2%

Type of tremor		

Postural	43	87.7%

Intentional	28	57.1%

Kinetic	13	26.5%

Rest	18	36.7%

Head tremor	27	55.1%

Voice tremor	21	42.8%

Jaw tremor	5	10.2%

Tongue tremor	5	10.2%

**History of Imbalance at presentation**	36	73.5%

Duration	4.88 ± 3.86 years	

Ataxia on examination	44	89.8%

Impaired tandem gait	44	89.8%

Dysmetria	37	75.5%

Dyssynergia	33	67.3%

Dysdiadokinesia	33	67.3%

Dysarthria	28	57.1%

Saccadic abnormality	13	26.5%

Broken pursuits	14	28.6%

Nystagmus	10	20.4%

Need for walking aid	8	18.2%

**Pyramidal system**		

Hyperreflexia	23	46.9%

Spasticity	9	18.4%

Extensor plantar	4	8.2%

**Extrapyramidal system**		

Rigidity	7	14.2%

Bradykinesia	15	30.6%

Chorea	1	2%

Dystonia	4	8.2%


Abbreviations: UL- Upper Limb, DTR- deep Tendon Reflexes.

### Investigations

#### Genetic results

The mean expanded CAG repeat length of *PPP2R2B* gene was 53.26 ± 6.10 repeats (median 52 repeats, range 40–72 repeats) while that of the shorter allele was 15 ± 7.13 (median 13, range 9–40). There was no significant correlation between number of expanded CAG repeats with age at onset or duration of illness (P > 0.05). The largest expanded repeat size observed in this cohort was 72 CAG repeats and the lowest repeat size was lower than 43 repeats. The details of these two patients are given below as case vignettes.

#### Radiological features

**Figure 3:**
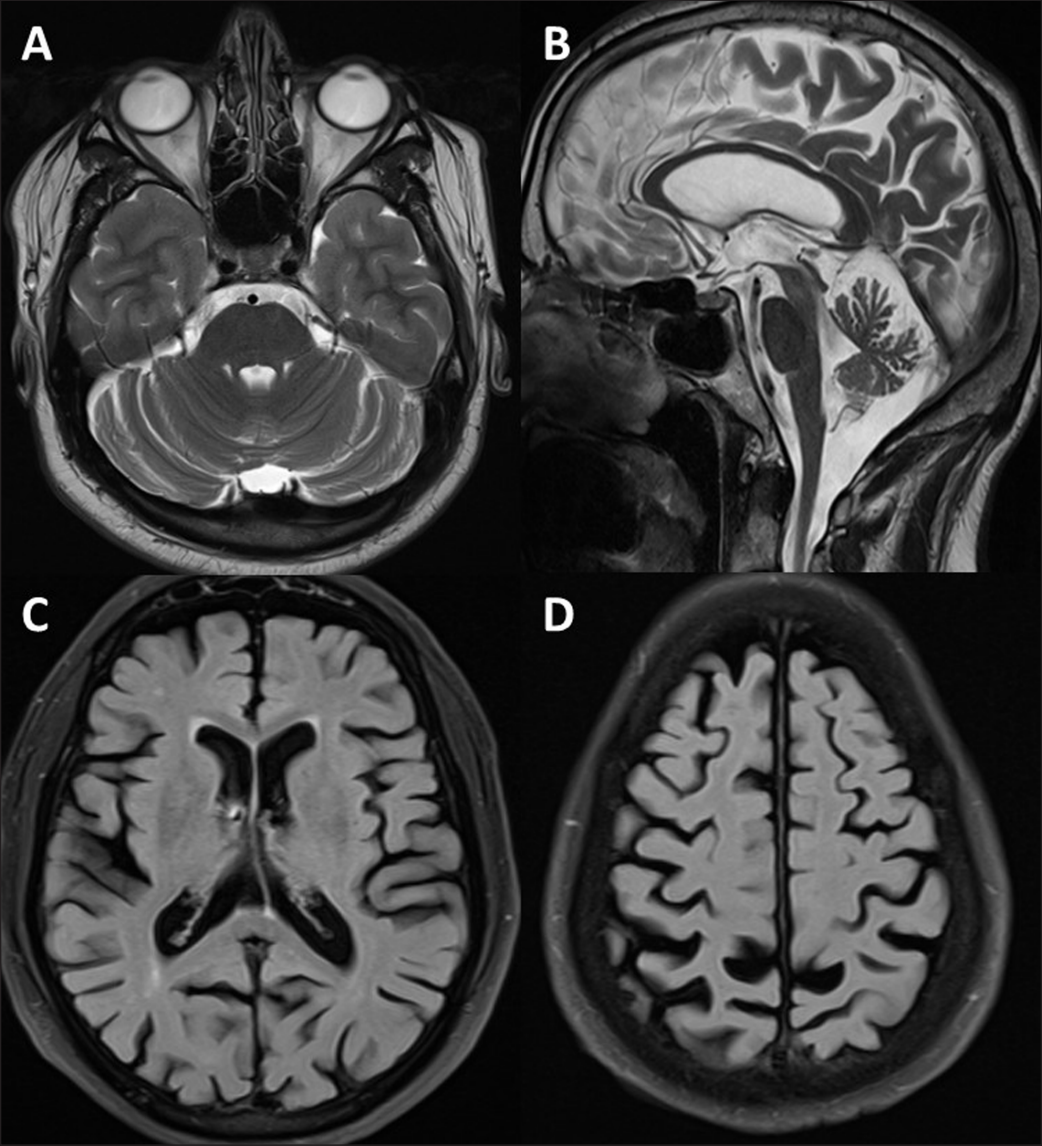
MRI brain showing cerebellar atrophy in (**A**) T2 axial and (**B**) mid-sagittal sections and cerebral atrophy in (**C, D**) FLAIR axial sections.

Magnetic resonance imaging of the brain was available for all the patients. Cerebellar atrophy was the sole imaging finding in 17 patients (34.7%) and eight patients (16.3%) only had cerebral atrophy (***Figure 3***). Concurrent cerebral and cerebellar atrophy was present in 17 patients (34.7%). Other MRI changes observed were bilateral basal ganglia mineralisation, and periventricular ischemic changes observed in 2 patients (4%) each. Normal brain imaging was observed in 3 patients (6.1%).

### Treatment

In the absence of specific disease targeting therapy, the patients were prescribed various medication based on the phenomenology. Nearly 73.5% of patients were treated with least two or more combination of medicines targeted for symptomatic control of tremors, ataxia, spasticity, rigidity and dystonia. Most commonly used medicines were amantadine (53%), propranolol (53%) clonazepam (41.8%), primidone (28.5%), L-Dopa (10.2%), trihexyphenidyl (6.1%) and baclofen (4%). Subjective improvement in symptoms such as tremor, ataxia was reported by the patient and their caregivers but there were no objective assessments to substantiate this. Details are as given in ***Table 3***.

**Table 3:** Profile of medications use among patients with SCA 12.


MEDICATIONS TRIED	NUMBER OF PATIENTS	PERCENTAGE (%)

Amantadine (100 – 300mg/day)	26	53%

Propranolol (20 – 60 mg/day)	26	53%

Benzodiazepines	21	42.8%

Primidone (250 – 500mg/day)	14	28.5%

L Dopa (400 – 600mg/day)	5	10.2%

Trihexyphenidyl (6 – 12 mg/day)	3	6.1%

Baclofen (20 – 40 mg/day)	2	4%

**COMBINATION OF MEDICINES**		

One	13	26.5%

Two	26	53%

Three	6	12.1%

Four	4	8.2%


### Case vignette

#### Case-1

A 42-year-old male from *Agarwal* family belonging to eastern part of India, presented with tremors in right hand while writing since the age of 41-years. On examination, he had bilateral postural tremors without any features of ataxia. His 67-year-old mother also had similar features of bilateral upper limb postural tremors of 17-years duration with late onset of cerebellar ataxia and voice tremors. His maternal uncle, maternal aunt and grandmother also had similar illness. He was diagnosed elsewhere as essential tremor. However, his MRI brain of the patient showed cerebral and cerebellar atrophy and a genetic screen for spinocerebellar ataxia subtypes showed a CAG repeat length of 42 repeats in *PPP2R2B* gene. Despite being in the range of intermediate expansion, the diagnosis was revised to SCA12. Subsequently, his mother’s and maternal uncle’s MRIs and genetic tests was performed. These investigations revealed cerebral and cerebellar atrophy with expanded CAG repeat size in pathological range.

#### Case-2

A 44-year-old female from *Agarwal* family belonging to eastern part of India, had features of imbalance since five years. It was accompanied by tremors in both hands while holding objects and while doing work. On examination, she had features of cerebellar ataxia with postural and intentional tremors involving both hands. His father and paternal grandfather also had features of similar illness. The MRI brain showed severe cerebellar atrophy with a CAG repeat length was 40 in *PPP2R2B* gene. Despite being in the intermediate range of expansion, the clinical picture and MRI in conjunction helped establish a diagnosis of SCA12.

#### Case-3

A 58-years old gentleman from *Agarwal* family presented with jerky movements of both upper limbs started at the age of 43 years old and was sometimes associated with dropping objects. In the same time, he developed imbalance while walking with frequent falls. His father also had features suggestive of tremors in hands and imbalance while walking, started by the age of 45 years. On examination, Tremor was present in both upper limbs mainly in posture with intermittent myoclonus. He also had incoordination in upper limbs along with ataxic gait and impaired tandem walking. There was flexion at wrists and hyperextension of metacarpophalangeal joints leading to “spooning” dystonia, which was more evident in outstretched hands (***Video 1***). His MRI brain showed cerebellar atrophy and a genetic screen for spinocerebellar ataxia subtypes showed a CAG repeat length of 54 repeats in *PPP2R2B* gene.

**Video 1:** **Video of Case-3.** Video showing postural tremors in both upper limbs with intermittent myoclonus, spooning dystonia over both hands and features of incoordination in upper limbs with ataxic gait and impaired tandem walking.

## Discussion

We report our experience with 49 patients of genetically proven SCA12. The mean age of onset our cohort was comparable to those reported in previous studies [4,5]. A male predominance was observed in our study in contrast to equal gender distribution noted in previous studies [4,5]. This gender difference is most likely due to the biased access to healthcare among male and female patients or may be due to the perceived occupation-related disability. In most patients, the presentation was tremor followed by ataxia. In this study, we report the presence of myoclonus, a previously unreported finding in SCA12. Furthermore, we also report two patients with intermediate CAG repeat expansion with 40 and 41 with classical clinical presentation of SCA12. Trinucleotide repeat lengths did no correlate with either age at onset or duration of illness.

As per existing literature, majority of the patients in our cohort belonged to *Agarwal* community. However, nearly 10% of the patients did not either disclose their community or were from non-*Agarwal* communities originating from southern part of India. Although the first description of SCA12 was from German ancestry [6], the first Indian origin case was described by Fujigasaki in 2001 [7]. Since then, many reports of SCA12 from northern part of India was described. Vast majority of patients with SCA12 reported from India belongs to an endogamous *Agarwal* ethnic group originating from north-eastern part of India which explains the majority of the reports from that part of India. A common founder mutation is implicated. However, SCA12 cases can be seen in other ethnicities with 5–8% cases belonging to non-*Agarwal* ethnicities according to various reports [5,8]. Five cases in this study had non-*Agarwal* ethnicity, three of which have been previously reported [9]. While it is difficult to study their ancestry, the non-confinement of SCA12 to *Agarwal* community is clinically relevant in India as it is not an uncommon practice to rule out SCA12 when the patient is from non-*Agarwal* communities in India. Often many of these patients are diagnosed as essential tremor (case vignette-1) and Parkinson’s disease. Clinicians should be aware that members from non-Agarwal communities may also develop SCA12 as a de-novo mutation, or more commonly due to events such as ancestral migration of population, inter-community marriages, disputed parentage, and adoption. Possibility of SCA12 should hence be considered in individuals who present with tremors and ataxia even among non- *Agarwal’s*.

In keeping with the clinical description, the most common symptom at the onset was tremor [3,4,5,10,11]. However, unlike some of the previous studies [4,5], the tremor remained the most common symptom even at the time of presentation as opposed to an ataxia predominant phenotype. The tremor was more often unilateral at onset, and as the disease progressed involved the other sides with the majority of the patients having bilaterally symmetric involvement at the time of presentation. In this study, asymmetric tremor was noted only in few patients. In contrast, in the study by Bhansali et al (2021), asymmetry was the rule even at the time of presentation with more than 90% cases having asymmetrical tremor which was most evident in postural component [12]. This difference may be attributed to the objective scale applied in the study by Bhansali et al which was lacking in the present study. Apart from the upper limb involvement seen in almost all the patients, other regions can also be involved with head tremor and voice tremor observed in around half of the cases and jaw and tongue tremor in a few patients [4]. Head tremor and voice tremor are frequently seen patients with SCA12 and is one of the classical features [4]. Based on the activation pattern, postural tremor was the most frequent type. Apart from this, Holmes’ tremor has also been described [12].

Ataxia was observed in close to 90% percent of the patients on examination even though only around 70% reported it as a symptom. This may be due to mild degree of ataxia unnoticed by the patients, ataxia attributed to the tremor, or overshadowing of ataxia symptoms in the background of severe disability due to tremor. All patients with ataxia on examination had impaired tandem gait with nearly two-third having dysarthria and nearly one-third having eye-movement abnormality. Ataxia is less severe compared to other trinucleotide repeat disorders with approximately one in five patients requiring walking aid at the time of presentation [5,13]. Cerebellar signs often become more prevalent and prominent as the disease progresses and overshadows tremor. Patient often have pan-cerebellar dysfunction with appendicular, axial, speech and eye movement abnormalities [5].

Apart from tremor and ataxia, patients may have other neurological involvement. To the best of our knowledge, myoclonus has never been reported in patients with SCA12 which was observed in 3 patients in the present study [14]. The myoclonus was multifocal and occurred in all four limbs suggesting a possibility of either cortical or subcortical origin. Cognitive dysfunction, behavioural abnormalities, pyramidal dysfunction, extrapyramidal involvement, peripheral nerve involvement and autonomic dysfunction can be seen in patients with SCA12 in variable frequency [5,11,15,16,17]. Dystonia was observed in only 8% cases in comparison to nearly two-third of cases in a previous study [5]. One of our patients with myoclonus, also had features of “spooning dystonia” a subtle form of dystonia, described previously in patients with essential tremors [18]. Previous case reports of atypical presentations such as atypical parkinsonism with abnormal DatScan [17], spasmodic dysphonia [19], and Huntington disease like presentations [16,20] are known. Hence a high index of suspicion is required for correct diagnosis.

The pathogenic repeat size CAG repeats of *PPP2R2B* gene ascribed to SCA12 is not very clear. Initially it was more than 51 which was subsequently reduced to 43. However, studies have demonstrated that clinical features of those with repeat lengths of 43–50 was comparable to those with 51 repeats. In the present study, two patients with typical SCA12 phenotype and positive family history had a repeat length of 40 and 41. There are other reports of unusual occurrences of SCA12 phenotypes with repeat lengths of 36–51 [4,21]. Although these repeat sizes are considered as intermediate repeats, it should be noted that these phenotypes are typical of SCA12. It is possible that pathological repeat size may be lower than previously suggested rather than these lower repeats regarded as intermediate repeats. However, to confirm these larger cohorts of patients with repeat sizes in the grey zone should be evaluated.

In contrast to other ataxias due to trinucleotide repeat expansion, the repeat lengths do not correlate well with the onset or the severity of the illness. Whereas one study [4] found no correlation between repeat length and the disease onset or severity, another study [5] had a contrasting finding with significant inverse correlation of the repeat length with age at onset but not with disease severity. We could not assess correlation between repeat length and severity as severity scores were not available in the majority of our patients. However, we did not find any significant correlation between the age at the onset of clinical symptoms and the CAG repeat size.

Imaging features in this study typically suggested mild to moderate cerebral and cerebellar atrophy in majority of patients with relative preservation of basal ganglionic structures and brainstem. Similar observations were made earlier [10]. Loss of Purkinje cells has been postulated to be the reason for cerebello-cortical degeneration [11]. Currently there is no definitive therapy for SCA 12 and therefore symptomatic treatment remains the mainstay of treatment. However, the response is often variable and unsatisfactory as evident by multiple medications tried for tremor in our cohort. Various classes of medications used for tremor have been used with variable results. As for tremor of other aetiologies, whether tremor of SCA12 responds to deep brain stimulation or lesioning surgeries is yet to be determined. There is one report of bilateral zona incerta DBS performed in a SCA12 patient with improvement in tremor scores [22]. However, at the stimulation parameters required to control tremor, there was worsening of ataxia and hence the patient could not benefit from the surgery.

A major limitation of this study was the retrospective nature and the lack of objective measures for assessing tremor type, degree and frequency along with ataxia and other non-ataxic features. Additionally, electrophysiological tests like tremor recording and neuropsychological assessment among these patients could give more insight into this autosomal dominant form of SCA subtype. In addition, owing to the retrospective nature, subtle movement disorders such as dystonia, chorea etc might have been underestimated and missed. Objective measures of symptomatic treatment outcome would have given more insight into this rare form of ataxia.

## Conclusion

Tremor followed by ataxia are the main symptoms at onset as well at presentation in patients with spinocerebellar ataxia type-12. Even though tremor is often unilateral at onset, it progresses to involve the opposite side and is often bilateral at the time of presentation. In a patient with otherwise typical presentation, SCA12 should be considered irrespective of the ethnicity and the geographical distribution as it need not be restricted to one particular community or a geographical distribution. Apart from limb tremor, head and voice tremor can be commonly seen. In addition to tremor and ataxia, patients with SCA12 can have pyramidal, extrapyramidal, cognitive and behavioural impairment. The causal repeat length can be as low as 40 and the repeat length may not correlate with the age at onset.
